# Does HFpEF Exist? A Comment on “The Concept of ‘Heart Failure with Preserved Ejection Fraction’: Time for a Critical Reappraisal” (Rev Cardiovasc Med. 2023;24(7):202)

**DOI:** 10.31083/RCM42408

**Published:** 2025-07-25

**Authors:** Maria Giulia Bellicini

**Affiliations:** ^1^Institute of Cardiology, ASST Spedali Civili di Brescia, 25123 Brescia, Italy; ^2^Department of Medical and Surgical Specialties, Radiological Sciences, and Public Health, University of Brescia, 25123 Brescia, Italy

Dear Editor,

I read with interest the recent article by Fragasso [1], which argues for a reappraisal of heart failure with preserved ejection fraction (HFpEF) as a multifactorial syndrome rather than a unified disease entity. While the author emphasizes the heterogeneity and overlapping comorbidities in patients diagnosed with HFpEF, I believe the issue extends further. At first glance, HFpEF might appear to represent a subclinical predisposition—an early-stage heart failure phenotype in structurally borderline cases. But a closer examination of the evidence base suggests a different picture: HFpEF is an abstract category born from classification need more than from clinical coherence. Much of the clinical reasoning and conceptual critique presented below has also been elaborated in a forthcoming commentary accepted for publication in *Internal and Emergency Medicine* [2].

Despite being framed as trials of a distinct clinical entity, many major HFpEF studies enrolled patients who were either not truly decompensated or did not meet criteria for pure HFpEF phenotype. In CHARM-Preserved [3] and Treatment of Preserved Cardiac Function Heart Failure With an Aldosterone Antagonist (TOPCAT) [4], PARAGON-HF [5] and DELIVER [6] severe valve disease and cardiomyopathy were formally excluded but echocardiogrm was not systematically performed or standardized to reliably exclude HFpEF mimics—such as valvular disease, cardiomyopathies, and pulmonary hypertension—and in some cases it is even unclear how preserved EF was documented.

Moreover the presence of heart failure was often inferred not from echocardiographic or radiographic congestion, nor from pulmonary artery pressure vale nor from diastolic pattern, but from international classification of diseases (ICD) codes or natriuretic peptide levels. Selected thresholds, however, were often within physiological ranges for elderly or acutely ill patients, especially those with atrial fibrillation. European Society of Cardiology (ESC) guidelines [7], for example, set the diagnostic n-terminal pro b-type natriuretic peptide (NT-proBNP) threshold at 1800 pg/mL for individuals over 75. Instead in TOPCAT [4], patients qualified via elevated BNP (≥100 pg/mL), NT-proBNP (≥360 pg/mL), or prior HF hospitalization, that, particularly in Eastern Europe, turned out to represent non-HF events. In later trials like PARAGON-HF [5], EMPEROR-Preserved [8], and DELIVER [6], HF was defined primarily by natriuretic peptide levels ≥300–600 pg/mL (≥900 in AF).

Furthermore, given the limitations of imaging technology in the early 2000s, it is likely that many cases of severe valve disease were not detected. Moreover, the limited recognition of wild-type transthyretin amyloidosis (ATTR), a condition now increasingly recognized, contributed to the creation of the “diastolic dysfunction in presence of hyperthrophy but without significant classical structural abnormalities” narrative, which in turn shaped the HFpEF concept as a distinct syndrome.

To illustrate this, we analyzed 773 consecutive admissions for acute heart failure (AHF) in our cardiology department, each evaluated with protocolized echocardiography upon arrival. Among 323 patients with left ventricular ejection fraction (LVEF) >40%, only 252 had confirmed echocardiographic congestion, of these last group, over 90% of patients had a clearly attributable causes of the following: severe valve disease (81.7%), cardiomyopathies, severe pericardial effusion, hemodynamically relevant rhythm disorders, or stage V kidney failure. Only 8% had no cardiac structural or extracardiac explanation.

These findings, publicly available, suggest that genuine ‘HFpEF-related’ decompensation in a heart free of HFpEF mimics is rare. Even so, the fact that severe functional mitral (and occasionally tricuspid) insufficiency often regressed at follow-up suggests that what is sometimes labeled as ‘pure’ HFpEF may actually reflect a subclinical phase of heart failure. However, I believe that these patients—when they do present with decompensation—are not representative of those typically enrolled in HFpEF trials, nor are they the patients described in current guideline definitions. In daily clinical practice, particularly in internal medicine wards, any patient presenting with dyspnoea may be labeled as HFpEF almost by default. In my experience as a consultant, HFpEF is often considered in elderly patients (with no prior history of heart failure, heart disfunction or severe valve disease) hospitalized with acute dyspnoea, even when an alternative cause—such as chronic obstructive pulmonary disease (COPD) exacerbation or pneumonia—appears more likely. This reflects the diagnostic complexity of acute settings, where overlapping symptoms can challenge certainty and sometimes prompt precautionary co-diagnoses. However, cardiac imaging at admission is frequently absent (whereas in my cohort, echocardiography was systematically performed upon arrival) and when cardiology evaluation is eventually performed, the cardiac picture is often within normal limits. This diagnostic shortcut may reflect the inherent uncertainty of acute care, yet it leads to a proliferation of erroneous HFpEF diagnoses.

Some have proposed mechanistic models—ranging from systemic inflammation to endothelial and microvascular dysfunction—as drivers of HFpEF via myocardial stiffening. Yet these theories, while intellectually attractive, remain largely unproven [9]. The clinical features often proposed as evidence for such cellular dysfunction—such as comorbidities including chronic kidney disease, atrial fibrillation, advanced age, hypertension, or obesity—are not, however, consistently associated with acute decompensation in patients with preserved ejection fraction (EF). For example, in the hypertensive heart disease phenotype, left ventricular hypertrophy, when not severe and not suggestive of cardiomyopathy, is rarely associated with decompensation—as confirmed in my daily practice. In atrial fibrillation, decompensation generally occurs either in the presence of pre-existing reduced EF or as a result of tachycardia-induced cardiomyopathy. Finally, the so-called cardiorenal HFpEF often reflects volume overload due to advanced renal failure rather than intrinsic cardiac dysfunction—something that typically becomes clinically evident only when the glomerular filtration rate falls below 15 mL/min [10]. Elderly were frequently hospitalized in internal medicine ward and of this thigh I have talked yet.

Fragasso calls for a pathophysiologic perspective [1], and I agree—but would go further: rather than viewing HFpEF as a real but poorly understood condition in need of mechanistic clarity, we should recognize that ‘pure’ HFpEF likely does not exist—not even as a subclinical syndrome. It is a retrospective construct, shaped by inclusion logic, not by physiologic definition. Reframing HFpEF as an abstract category could restore diagnostic rigor and reduce therapeutic overreach especially in evaluation of acute patient. Moreover, some might argue that therapeutic advances—such as sodium-glucose cotransporter-2 (SGLT2) inhibitors—have shown benefit even in such heterogenous groups, which could be seen as validation of the HFpEF concept.

## Abbreviations

AHF, Acute Heart Failure; ATTR, Transthyretin Amyloidosis; COPD, Chronic Obstructive Pulmonary Disease; EF, Ejection Fraction; ESC, European Society of Cardiology; HF, Heart Failure; HFpEF, Heart Failure with Preserved Ejection Fraction; ICD, International Classification of Diseases; LVEF, Left Ventricular Ejection Fraction; NT-proBNP, N-terminal pro b-type Natriuretic Peptide; TOPCAT, Treatment of Preserved Cardiac Function Heart Failure With an Aldosterone Antagonist.
